# The interaction of mast cells with membranes from lung cancer cells induces the release of extracellular vesicles with a unique miRNA signature

**DOI:** 10.1038/s41598-023-48435-4

**Published:** 2023-12-06

**Authors:** Rachel Shemesh, Smadar Laufer-Geva, Yaara Gorzalczany, Alaa Anoze, Ronit Sagi-Eisenberg, Nir Peled, Laila C. Roisman

**Affiliations:** 1 Department of Cell and Developmental Biology, Faculty of Medicine, Tel Aviv University, Tel Aviv, Israel; 2 Goldschleger Eye Institute, Sheba Medical Center, Tel-Hashomer, Israel; 3 Edmond and Lily Safra Children’s Hospital, Sheba Medical Center, Ramat Gan, Israel; 4 The Helmsley Cancer Center, Shaare Zedek Medical Center, Shmu’el Bait St 12, Jerusalem, Israel; 5 The Hebrew University of Jerusalem, Jerusalem, Israel

**Keywords:** Cancer, Immunology

## Abstract

Mast cells (MCs) are immune cells that play roles in both normal and abnormal processes. They have been linked to tumor progression in several types of cancer, including non-small cell lung cancer (NSCLC). However, the exact role of MCs in NSCLC is still unclear. Some studies have shown that the presence of a large number of MCs is associated with poor prognosis, while others have suggested that MCs have protective effects. To better understand the role of MCs in NSCLC, we aimed to identify the initial mechanisms underlying the communication between MCs and lung cancer cells. Here, we recapitulated cell-to-cell contact by exposing MCs to membranes derived from lung cancer cells and confirming their activation, as evidenced by increased phosphorylation of the ERK and AKT kinases. Profiling of the microRNAs that were selectively enriched in the extracellular vesicles (EVs) released by the lung cancer-activated MCs revealed that they contained significantly increased amounts of miR-100-5p and miR-125b, two protumorigenic miRNAs. We explored the pathways regulated by these miRNAs via enrichment analysis using the KEGG database, demonstrating that these two miRNAs regulate p53 signaling, cancer pathways, and pathways associated with apoptosis and the cell cycle.

## Introduction

During the past decade, there have been substantial advancements in therapeutic options for lung cancer, especially adenocarcinoma. New oral agents were approved as first-line or advanced therapies, as targeted therapies for patients with specific targetable mutations, or as immune checkpoint inhibitors and their different combinations^1^. Gaining insight into the molecular pathways that dictate the response to immunotherapies remains a pivotal obstacle in the pursuit of precise immuno-oncology approaches.

The interaction between cells and their microenvironment is a crucial factor in maintaining a normal tissue balance and promoting tumor development. As environmental factors evolve and tumors produce oncogenic signals, the tumor microenvironment (TME) undergoes continual changes during cancer progression^2^.

The TME contains a variety of noncancer cells, including inflammatory cells such as dendritic cells and mast cells (MCs). These nontumor cells play significant roles within the TME and contribute to the complex interplay between cancer cells and their surroundings^3^. MCs are secretory cells that are best known for their role in allergy, but they also have a protective role in immune defense. MCs are involved in tissue remodeling, wound healing, and protection against pathogens. MCs are characterized by “plasticity”, which can result in the development of phenotypically distinct populations of MCs at different anatomic sites^4^. This plasticity could be specifically important in the context of tumors, where MC phenotypes and functions—which can vary from protumorigenic to antitumorigenic—may be influenced by the TME and may alter during the evolution of the disease^5^. Notably, MCs accumulate within solid tumors and can stimulate tumor progression and metastasis via the release of angiogenic factors or growth-promoting factors^6^. A high density of MCs within the TME has been linked to unfavorable prognoses, heightened metastatic potential, and reduced survival rates across several types of cancers, including lung cancer^7,8^. Moreover, MCs were found to have a direct growth-promoting effect on non-small cell lung cancer (NSCLC) cells^9^. Exosomes are nanosized extracellular vesicles that participate in cell-to-cell communication through the transfer of biomolecules from one cell to another^10^. Specifically, the cargo of tumor-derived exosomes is enriched in molecules, such as microRNAs (miRNAs), which remodel the TME and promote cancer development. miRNAs are small noncoding molecules that are involved in the regulation of multiple cellular roles, such as differentiation, proliferation, and apoptosis, via the posttranscriptional regulation of gene expression^11,12^. The abnormal expression of specific miRNAs can therefore result in the dysregulation of signaling components and plays a major role in tumor cells^13^.

Previously, we and others have demonstrated the intercommunication between cancer cells and MCs, where lung cancer cells release extracellular vesicles that can activate MCs, and conversely, MC exosomes were shown to transfer soluble protumorigenic factors to lung cancer cells that accelerated their proliferation^10,14–16^. Thus, we explored the possibility that MC activation by lung cancer cells influences the miRNA cargo of the exosomes to be released by the activated MCs. Here, we present the results of miRNA profiling of MC-derived EVs and the associated bioinformatics analyses.

## Results

### Mast cell activation by lung cancer-derived membranes

**Figure 1:**
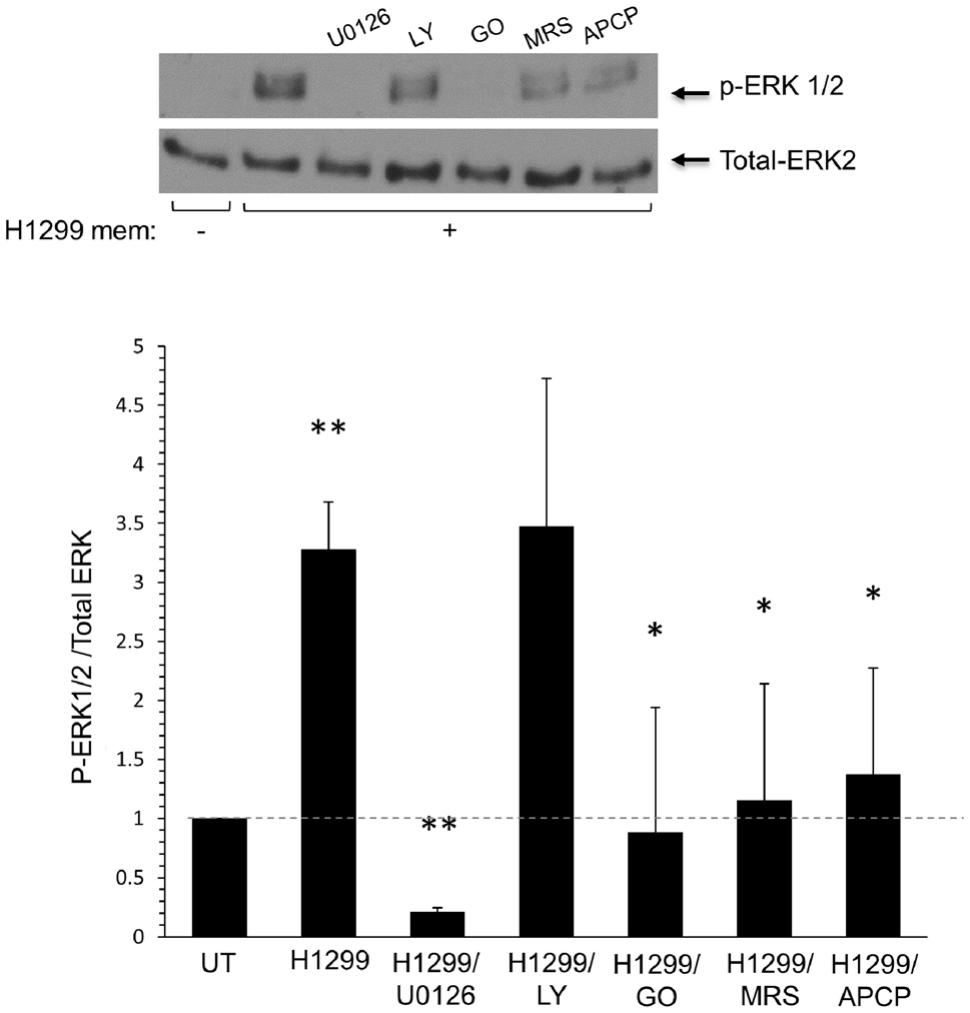
The phosphorylation of ERK 1/2 in HMC-1 cells stimulated by H1299-derived membranes was evaluated upon inhibition of MEK, PI3K, PKC, A3R, and CD73. HMC-1 cells (1 × 10^6^ cells/ml) were pretreated with the indicated inhibitors (3 µM U0126, 10 µM LY 294002, 1 µM GO 9676, 100 nM MRS 1220, and 5 µM APCP, respectively) for 30 min. Subsequently, the cells were exposed to 10 µg/ml H1299-derived membranes for 1 min. Cellular lysates underwent SDS-PAGE separation and were subjected to probing with an anti-p-ERK 1/2 antibody and subsequent reprobed using an anti-total ERK2 antibody. The phospho-ERK 1/2 and total ERK bands were determined using ImageJ software, and the relative pixel densities (phosphorylated/total) were calculated and normalized to those of untreated (UT) cells. The data presented reflect the average of three distinct experiments and are shown as the mean ± SEM (error bars). A *p* value less than 0.05 was considered significant and denoted by *, while ** indicates a *p* value less than 0.01.

**Figure 2:**
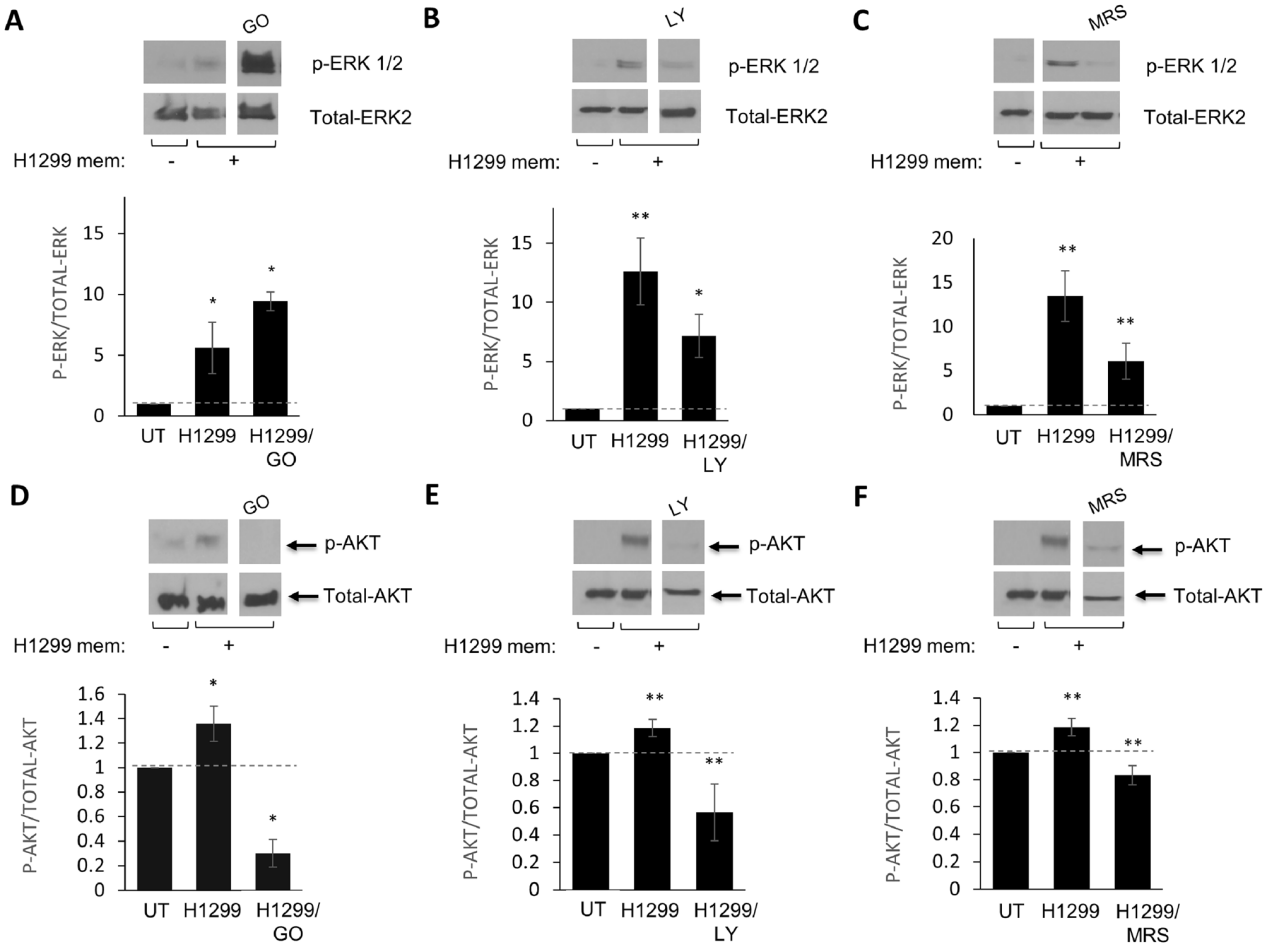
The impact of inhibiting PI3K, PKC and A3R on the phosphorylation of ERK 1/2 in LAD-2 cells stimulated by H1299-derived membranes was assessed. LAD-2 cells were subjected to a 30-min preincubation with vehicle (UT) (**A,D**), the PKC inhibitor Go6976 (1 µM), (**B,E**) the PI3K inhibitor LY 294002 (10 µM), or (**C,F**) the A3R inhibitor MRS 1220 (100 nM). The cells were then immunoblotted with an anti-p-ERK 1/2 antibody, followed by reprobing with an anti-total ERK2 antibody (**A–C**), or with an anti-p-AKT antibody, followed by reprobing with an anti-total AKT antibody (**D–F**). The data presented reflect the average of three distinct experiments and are shown as the mean ± SEM (error bars). A *p* value of less than 0.05 was considered significant and indicated by *, while ** indicates a *p* value of less than 0.01.

Previously, we showed that coculture of human MC lines (HMC-1 and LAD-2 cells) and membranes derived from numerous cancer cell lines to recapitulate cell-to-cell contact^17^ causes MC activation, as indicated by increased phosphorylation of the MAP kinases ERK1/2 and AKT^16^. Consistent with our previous findings, we show here that the exposure of either HMC-1 (Fig. 1) or LAD-2 cells (Fig. 2) to membranes derived from the H1299 NSCLC cell line resulted a significant increase in the phosphorylation of ERK1/2 (*p* < 0.05), and that this effect was significantly inhibited by the MEK inhibitor U0126 (*p* < 0.01). Moreover, consistent with our previous results, which demonstrated the involvement of autocrine adenosine signaling in the cancer cell-mediated activation of MCs^16^, the present study shows that exposure to MRS1220, an antagonist of the adenosine A3 receptor significantly reduced ERK1/2 phosphorylation in both MC lines (Fig. 2). Interestingly, distinct sensitivities of the two human mast cell lines to LY294002, an inhibitor of PI-3 kinases, were also noted, where LY294002 inhibited ERK1/2 phosphorylation in LAD-2 cells but had no effect on ERK1/2 phosphorylation in HMC-1 cells (Fig. 1). Finally, most striking were the different responses of the cell lines to the drug Go6976, an inhibitor of classical protein kinase C (PKC) isoforms that significantly inhibited ERK1/2 phosphorylation in HMC-1 cells but significantly stimulated ERK1/2 phosphorylation in LAD-2 cells (Fig. 2). The reason for this discrepancy is presently unknown, although it may be related to the fact that in LAD-2 cells but not HMC-1 cells, exposure to H1299-derived membranes also results in increased phosphorylation of AKT, which might be inhibited by either LY294002 or Go6976 (Fig. 2).

#### miRNA profiling of MC EVs

Having confirmed the activation of MCs by H1299-derived membranes, we next analyzed the miRNA profile of EVs released from H1299-activated HMC-1 cells. We used the HTG EdgeSeq system and probed 2066 miRNAs. Following normalization and differential expression analyses, we found that three miRNAs—miR-31-5p, miR-100-5p, and miR-125b—were significantly upregulated in EVs released by H1299-activated MCs (Supplementary Table 1).

#### Validation of the upregulated miRNAs

**Figure 3:**
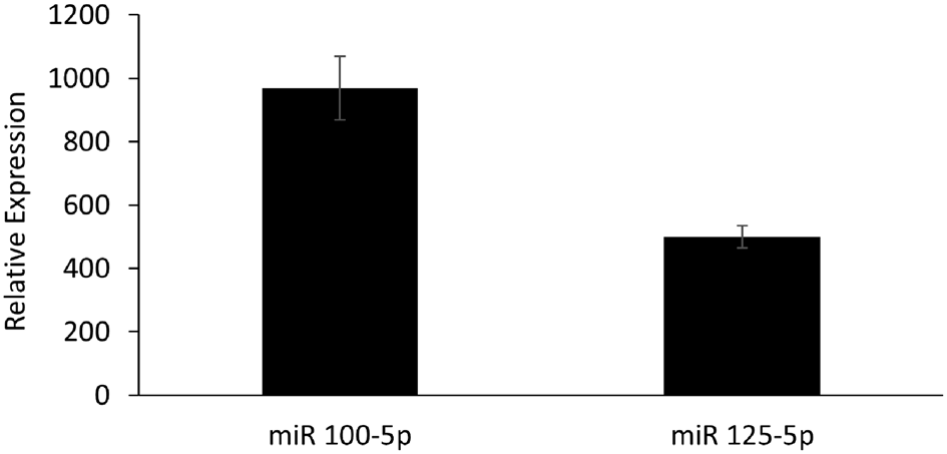
Quantitative real-time PCR (qRT‒PCR) validation of the presence of miR 100-5p and 125-5p in extracellular vesicles (EV) from HMC cells activated with H1299 membranes for 24 h compared to EVs from control HMC cells (untreated). The values shown were normalized to U6 snRNA as an endogenous control. The data are presented as the fold change in miRNA levels in treated vs. control exosomes and the mean ± SEM of 2 independent experiments.

To validate the results obtained with the array, we subjected the RNA derived from the spontaneously released EVs and EVs released by the HMC-1 cells that were activated by H1299 cell-derived membranes to quantitative real-time PCR using primers specific for each of our candidate miRNAs. The results of these analyses confirmed the exclusive presence of both miR-100-5p and miR-125b in the EVs released by the activated cells. In contrast, we could not validate the presence of miR-31-5p in the EVs released by the activated cells (Fig. 3).

#### Target analysis of miRNAs present in EVs from activated cells

**Figure 4:**
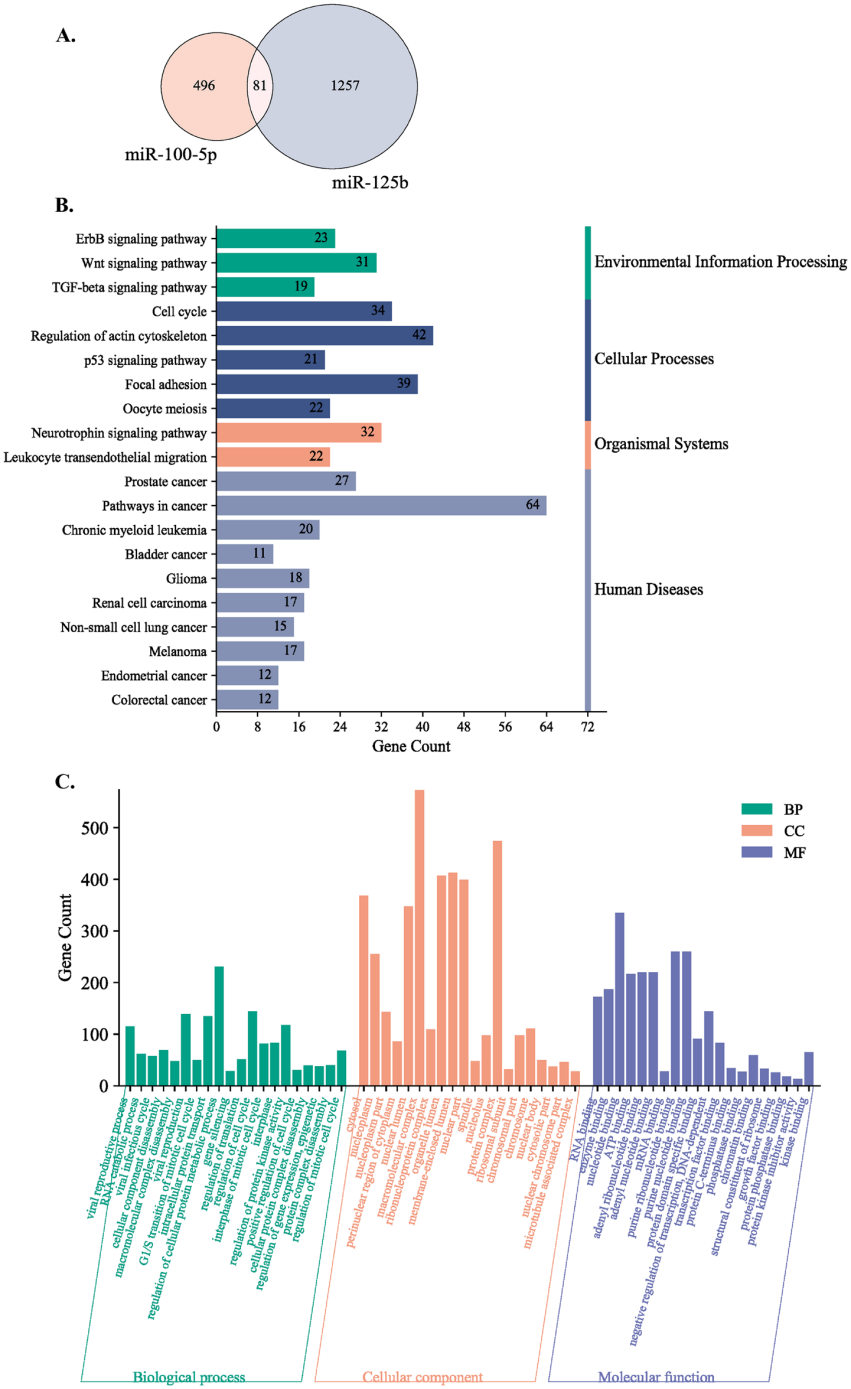
(**A**) Venn diagram of all genes regulated by the identified miRNAs. Bar plot of the top 20 enriched, (**B**) KEGG and (**C**) GO pathways for all target genes regulated by miR-100-5p and miR-125b.

To acquire a deeper understanding of the potential functions of the upregulated miRNAs, we used the miRTarBase^18^, TarBase v8^19^, and StarBase v2^20^ databases to uncover the target genes that are known to be regulated by miR-100-5p and miR-125b. The analysis revealed a total of 1915 target genes regulated by miR-100-5p or miR-125b (Fig. 4A). Specifically, mir-100-5p putatively regulates 577 target genes, mir-125b putatively regulates 1338 target genes, and 81 target genes are common to both miRNAs. Among the target genes common to the two miRNAs are AKT1, PIK3CB and BCL2, which are involved in numerous cancer pathways, with roles in apoptosis inhibition, sustained angiogenesis, and the promotion of tissue invasion and metastasis (Supplementary Table 2).

To better understand the functions carried out by the target genes under the regulation of these two miRNAs, we conducted a pathway analysis that encompassed all the relevant target genes. This analysis was performed utilizing the KEGG, GO:BP, GO:CC, and GO:MF databases. The 20 top pathways revealed by these analyses included pathways such as the p53 signaling pathway, cancer pathways, the cell cycle, the WNT signaling pathway, and focal adhesion pathways (Fig. 4B,C).

**Figure 5:**
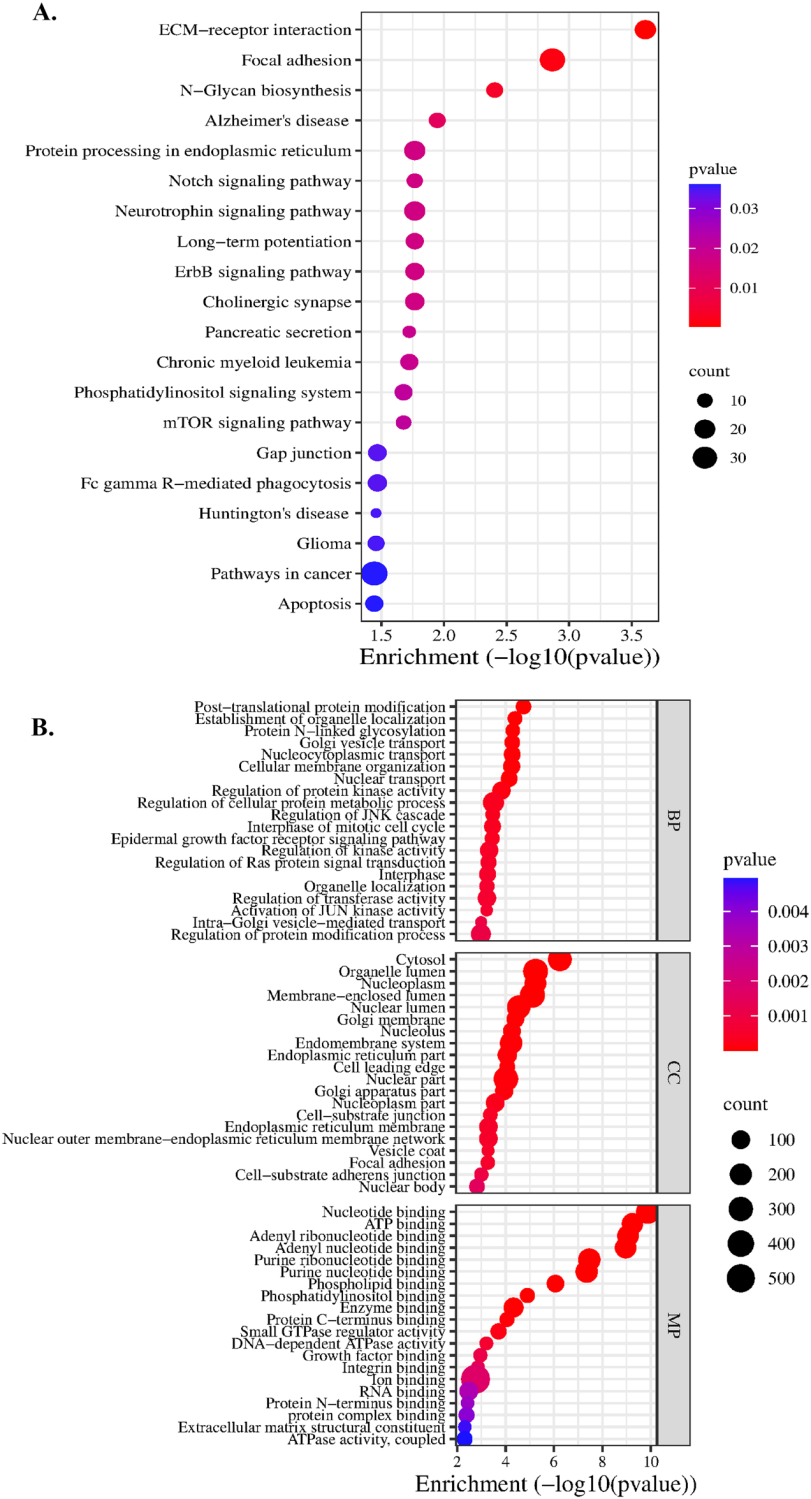
Bubble plot of the top 20 enriched (**A**) KEGG and (**B**) GO pathways activated by circRNAs interacting with miR-100-5p and miR-125b.

We also analyzed the potential interactions of the upregulated miRNAs with circular RNAs (circRNAs). This analysis revealed 130 circRNAs that are regulated by miR-100-5p and 1353 circRNAs that are regulated by miR-125b (Supplementary Table 3). Analysis of the pathways activated by those circRNA interactions using the KEGG database identified the mTOR signaling pathway, ECM–receptor interaction, epidermal growth factor receptor (EGFR) signaling pathway, cancer pathways, and genes involved in the regulation of focal adhesion and the cell cycle (Fig. 5).

Finally, an analysis of the potential interactions of miR-100-5p and miR-125b with long noncoding RNAs (lncRNAs) revealed that 8 lncRNAs interact with miR-100-5p and 47 lncRNAs interact with miR-125b, while Long Intergenic Non-Protein Coding RNA 943 (LINC00943) interacts with both miRNAs (Supplementary Table 4).

## Discussion

In this study, we explored the communication between mast cells and lung cancer cells through two aspects of the crosstalk between these cell types. We examined the activation of MCs by lung cancer cell membranes and analyzed the miRNAs in EVs released by lung cancer-activated MCs. First, we corroborated our previous findings that implicated CD73 and adenosine in MC activation by cancer cells^14,21^. Thus, extracellular adenosine not only inhibits immune responses in the TME^22^ but also contributes to the activation of MCs and thereby induces angiogenesis. Furthermore, strategies targeting the adenosine pathway are now available for NSCLC treatment. Examples include anti-CD73 antibodies combined with traditional cancer treatments (i.e., chemotherapy, radiotherapy, immunotherapy, and targeted therapy)^23^, such as the anti-CD73 antibody BMS-986179 in combination with nivolumab (NCT02754141)^24^, the anti-CD73 antibody MEDI9447 (oleclumab) in combination with the third-generation EGFR tyrosine kinase inhibitor (TKI) osimertinib or the selective adenosine 2A receptor (A_2_AR) antagonist AZD4635 (NCT03381274)^25^, and oleclumab in combination with durvalumab (NCT02503774)^26^, which are currently in phase 1 and 2 clinical trials. Such combinations may have the advantage of targeting not only the tumor but also its surrounding cells, including protumorigenic MCs.

Second, we analyzed the miRNA cargo of EVs released by MCs. We^14^ and others^27^ have demonstrated that EVs released by H1299 cells can activate MCs. Therefore, we analyzed whether the EVs that are released by H1299-activated MCs have modified miRNA contents. Although this issue is beyond the focus of the present study, it is anticipated that by modifying the miRNA content of the MC EVs, the tumor might exploit the MCs to change its own protein expression profile, enhancing its tumorigenic features. Therefore, identification of the specific miRNAs that are either upregulated or downregulated during MC-lung cancer crosstalk may offer a potential strategy for miRNA-based personalized therapy. This notion is particularly relevant to lung cancer, where the detection of circulating miRNAs showed that the levels of several miRNAs, such as *miR-122* and *miR-21*, are predictors of *EGFR* mutation status and efficiency of TKI in patients with NSCLC^28–32^.

In addition, various miRNAs have been found to target key cancer-related immune pathways, such as pathways involved in immune cell development and function^33^. The identification and characterization of miRNAs linked to genetic aberrations and immune responses may create new opportunities for tailored treatments and prognostic biomarkers in patients with NSCLC. Here, we identified two miRNAs, miR-100-5p and miR-125b, that are specifically enriched in EVs released by H1299-activated MCs. Numerous genes have been confirmed to be downstream targets of these miRNAs, such as the Class IV PI3K mTOR, the tyrosine kinase receptor MET, and the serine/threonine kinase and primary mediator of the PI3K signaling cascade AKT1, all of which are involved in cancer-related phenotypes^34,35^.

Consistent with these findings, analyzing the pathways targeted by these miRNAs using the KEGG pathway database revealed their connection to pathways involved in tumor phenotype, such as ERK, AKT, WNT, and ErbB pathways. These pathways contribute to oncogenic pathways such as apoptosis, proliferation, cell cycle, differentiation, angiogenesis, metastasis, and therapy resistance, suggesting that these miRNAs may play an important role in the regulation of NSCLC. Notably, miR-100-5p and miR-125b have previously been detected in various cancers and have been demonstrated to be involved in tumorigenesis^36^ and the cell cycle, including in NSCLC^37–42^.

Both miR-100-5p and miR-125b have been suggested as therapeutic targets for multiple types of cancers. Circulating miR-100-5p has previously been suggested as a potential biomarker for colorectal cancer^43^, prostate cancer^44^ and renal cell carcinoma^45^, while miR-125b has previously been suggested as a biomarker for breast cancer^46^ and stage I lung adenocarcinoma^47^ and is implicated as an oncogene or tumor suppressor in various other cancers owing to its role in regulating numerous signaling pathways^48,49^. Furthermore, miR-100-5p was shown to confer resistance to TKIs in *EML4-ALK* NSCLC cells^41^ and to be involved in promoting chemoresistance^50^. In addition, both miR-100-5p and miR-125b are associated with resistance to immunotherapy in patients with melanoma^51^. High levels of miR-125 were associated with unfavorable clinical outcomes in patients with NSCLC, with poorer chemotherapy responses, worse overall survival, and higher metastatic risk^52,53^. In addition, miR-125b plays a crucial role in the immune response by modulating^54–56^ T-cell proliferation and activation, inhibiting T-cell differentiation into effector cells, and promoting T-cell apoptosis^54,57^.

Lastly, our results suggest that AKT1 is part of the signaling cascade that is activated in H1299-activated LAD-2 cells. Moreover, we suggest that both the miRNAs could regulate ERK and AKT pathways via the regulation of key genes involved in these pathways. AKT1, a serine/threonine kinase, plays a well-documented role in numerous cellular processes and normal physiological development, and is particularly well known for regulating cellular growth, metabolism, survival, and proliferation^58^. Similarly, ERK has been described as a “master regulator” of cellular processes, both normal and aberrant^58^. The dysregulation of ERK, a member of the mitogen-activated protein kinase (MAPK) family, and AKT signaling has been implicated in a wide range of cancers, where the cross-talk between the PI3K/AKT and MAPK/ERK pathways has been associated with drug resistance^59^. Furthermore, both miR-100-5p and miR-125b have been shown to regulate AKT1 and PIK3CB^34,35^, while miR-125b is known to regulate EGFR, RAF1, and MAPK14^60,61^. Notably, while miR-125b has been shown to upregulate signaling pathways that are commonly activated in numerous cancers, such as Wnt/β-catenin and PI3K/AKT^49^ miR-125b has also been shown to suppress the progression of Ewing’s sarcoma, bladder cancer, gastric cancer, cervical cancer, thyroid cancer, and NSCLC via PI3K/AKT signaling pathways^35,62–66^. Treatments that include inhibitors of both PI3K/AKT and MAPK/ERK signaling pathways^59^ and/or treatments that target the miRNAs that regulate the downstream targets involved in these well-known signaling pathways hold considerable promise for treating various cancers.

In conclusion, our results further strengthen our proposed model, in which the activation of MCs by lung cancer cell-derived membranes is mediated by the phosphorylation of the ERK and AKT kinases^14,21^. We show here that the signaling cascades that are initiated in response to MC contact with lung cancer cell membranes lead to the production of two miRNAs, miR100-5p and miR125b, which are packaged into EVs that are released by activated MCs. Previous studies suggest that these miRNAs play both oncogenic and tumor suppressive roles indicating that future research is needed to further understand the crosstalk between lung cancer cells and MCs, as well as the role of miRNAs, to support the identification of new therapeutic targets that can inhibit this crosstalk and improve the prognosis of lung cancer patients.

## Materials and methods

### Materials

The A3 receptor antagonist 9-chloro-2-(2-furanyl)-5-(phenylacetyl) amino)-[1, 2, 4] triazolo [1,5 c] quinazoline (MRS 1220) was purchased from Sigma‒Aldrich. Bradford protein assay, Bio-Rad Laboratories. The PKC inhibitor GO6976, PI3K inhibitor LY294002, MEK inhibitor U0126, and CD73 inhibitor APCP were purchased from A.G. Scientific Inc. The antibodies used in this study included mouse polyclonal anti-phospho-ERK 1/2 (Sigma Aldrich), rabbit polyclonal anti-ERK2 (Santa Cruz Biotechnology Inc.), polyclonal anti-phospho AKT, Ser473 and polyclonal anti-AKT (Cell Signaling, Beverly, MA) antibodies.

### Cell culture

The HMC-1 human mast cell line (gift from Joseph H. Butterfield, Mayo Clinic) cultured in RPMI 1640 medium supplemented with 10% fetal bovine serum (FCS), 2 mM glutamine, 100 µg/ml streptomycin, 100 units/ml penicillin, and 12.5 units/ml nystatin. The LAD-2 human mast cell line (gift from Arnold S. Kirshenbaum and Dean D. Metcalfe, NIH) was cultured in StemPro medium supplemented with 100 ng/ml hrSCF, 2 mM glutamine, 100 µg/ml streptomycin, and 100 units/ml penicillin. The H1299 NSCLC (American Type Culture Collection, ATCC) cell line was grown in DMEM supplemented with 10% FCS, 2 mM glutamine, 100 µg/ml streptomycin, 100 units/ml penicillin, and 12.5 units/ml nystatin. All cell lines were supplemented with 10% fetal bovine serum (FBS) that was depleted of exosomes (extracellular vesicles) via ultracentrifugation at 120,000×*g* overnight. The cells were maintained in a humidified atmosphere of 5% CO2 at 37 °C.

### Cancer cell membranes

Lung cancer cell membranes were prepared as described previously^21^. In brief, the H1299 lung cancer cell line was cultured in 10 cm^2^ plates and subsequently harvested. The cells were rinsed three times with PBS and then suspended at a concentration of 1 × 10^7^ cells/ml in ice-cold KTM buffer [50 mM Tris–HCl, pH 7.4, 25 mM KCl, 5 mM MgCl_2_, 250 mM sucrose, 1 mM PMSF, and a protease inhibitor cocktail (Boehringer Mannheim)]. The cell suspension was kept on ice for 20 min, followed by five cycles of freezing and thawing in liquid nitrogen to disrupt the cells. The mixture was centrifuged at 800×*g* for 5 min, and the supernatants were collected. Further centrifugation was performed at 37,000 rpm for 60 min at 4 °C using a Beckman centrifuge with a Ti-70 rotor. The resulting pellets, which contained the isolated membranes, were resuspended in PBS, divided into aliquots, and stored at − 80 °C.

### MC activation

Following 18–20 h of serum starvation, MCs were subjected to two washes with Tyrode buffer (137 mM NaCl, 2.7 mM KCl, 1 mM MgCl_2_, 1 mM CaCl_2_, 5.6 mM glucose, 1 mg/ml BSA, and 10 mM HEPES, pH 7.4). Subsequently, the MCs were transferred to Eppendorf tubes and incubated at 37 °C. Prior to further experimentation, the cells were preincubated with the specified inhibitors (3 µM U0126, 10 µM LY 294002, 1 µM GO 9676, 100 nM MRS 1220, or 5 µM APCP) for 30 min. Next, the MCs were incubated with H1299-derived membranes at a concentration of 10 µg/ml for 1 min. The reactions were halted by placing the tubes on ice. The cells were washed once with phosphate-buffered saline (PBS), followed by centrifugation at 1700×*g* for 5 min, and the resulting pellets were lysed using 35 µl of lysis buffer [50 mM HEPES, pH 7.4, 150 mM NaCl, 1% Triton X-100, 0.1% SDS, 50 mM NaF, 10 mM sodium pyrophosphate, 10 mM EDTA, 2 mM EGTA, 2 mM sodium orthovanadate, 1 mM PMSF, and a protease inhibitor cocktail (Boehringer Mannheim)]. The lysates were then incubated on ice for 10 min and subsequently centrifuged at 12,000×*g* for 15 min. The resulting cell extracts were mixed with 5× concentrated Laemmli sample buffer, boiled, and subjected to western blotting.

### Extracellular vesicle isolation

Cells were cultured at a density of 0.5 × 10^6^ cells/ml in 20 ml of medium for 24 h, and the resulting cell culture conditioned medium (CCM) was collected. To remove cells, the CCM was centrifuged at 1200 rpm for 5 min and then transferred to a new tube. Subsequently, the CCM was subjected to centrifugation at 4500×*g* for 5 min to eliminate large debris. The remaining supernatant was further processed by centrifugation at 100,000×*g* (37,000 rpm) for 70 min at 4 °C using a Beckman ultracentrifuge to isolate extracellular vesicles. These extracellular vesicles were washed with 20 ml of PBS and subjected to another round of centrifugation at 100,000×*g* for 70 min at 4 °C. Finally, the extracellular vesicles were resuspended in 50–100 µl of PBS and stored at − 80 °C for future use. Extracellular vesicles were isolated separately from each culture, including a culture of the human mast cell line HMC-1, a culture of the NSCLC cell line H1299, and a coculture of both cell lines. Furthermore, extracellular vesicles were also obtained from MCs that were activated by lung cancer cell-derived membranes.

### Isolation of total RNA and quantitative RT-PCR

Total RNA isolation was carried out utilizing the RNeasy Mini Kit (Qiagen, Hilden, Germany) following the protocols provided by the manufacturer. Subsequently, cDNA was synthesized from total RNA at a concentration of 2 ng/µl in a total volume of 15 µl using the High-Capacity cDNA RT Kit (Applied Biosystems). Quantitative real-time PCR was performed on an ABI Prism 7900 Sequence Detection System (Applied Biosystems) using TaqMan Fast Advanced Master Mix (Applied Biosystems). The obtained results were analyzed using Step Software v2.3 (Life Technologies). The relative levels of miRNA were determined employing the comparative threshold cycle (Ct) methodology. The Ct values of individual miRNAs and the endogenous U6 snRNA control within each sample were employed to compute ΔCt values (Ct miRNA–Ct U6 snRNA). Furthermore, ΔΔCt values were determined by deducting the ΔCt of the control group (exosomes from untreated HMC cells) from the ΔCt value of the experimental group (exosomes from HMC cells activated with H1299 membranes). The expression of specific miRNAs was assessed using the comparative Ct method, represented as 2-ΔΔCt. The following primers were employed: hsa-miR-100-5p MIMAT0000098-AACCCGUAGAUCCGAACUUGUG and hsa-miR-125b-5p MIMAT0000423-UCCCUGAGACCCUAACUUGUGA. U6 snRNA served as the endogenous control.

### miRNA sample preparation and miRNA profiling

For profiling of the miRNA content of EVs, total RNA was isolated from EVs released spontaneously by HMC-1 cells (3.5 × 10^7^ cells) or from HMC-1 cells (3.5 × 10^7^ cells) that were activated by H1299-derived membranes. RNA was analyzed by the HTG EdgeSeq system. Results are based on the analyses of three samples for each condition, derived from two independent experiments.

Exosome samples were purified were HTG EdgeSeq miRNA WT Assay. Samples were spun at 17,000×*g* for 2 h at 4 °C to pellet exosomes. Without disturbing the pellet, supernatant was removed and 30 μL of HTG Lysis Buffer was added to each exosome pellet. To each cell line sample, 2875 μl of HTG Lysis Buffer was added. Samples were then incubated at 95 °C for 15 min. To improve sample lysis, 1.5 μL of Proteinase K was added to each exosome sample. All lysates were incubated at 50 °C for 180 min after the addition of Proteinase K. Samples were run on an HTG EdgeSeq Processor using the HTG EdgeSeq miRNA WT assay. Following the processor step, samples were individually barcoded (using a 16-cycle PCR reaction to add adapters and molecular barcodes). Barcoded samples were individually purified using AMPure XP beads and quantitated using a KAPA Library Quantification kit.

The sample library was sequenced on an Illumina MiSeq using a V3 150-cycle kit with two index reads. PhiX was spiked into each library at 5%; this spike-in control is standard for Illumina sequencing libraries.

### Data presentation

A two-tailed Student’s t test was utilized for statistical analysis involving unpaired data. The presented data reflect the mean ± SEM and are the culmination of results from a minimum of three independent experiments. Significance was represented by *, indicating a p value of < 0.05, and ** denoted a p value of < 0.01.

### Supplementary Information

Supplementary Information.

## Data Availability

The datasets used and/or analyzed during the current study are available from the corresponding author on reasonable request.
