# Supplementary material for: The interaction of mast cells with membranes from lung cancer cells induces the release of extracellular vesicles with a unique miRNA signature
Source: Sci Rep. 2023 Dec 6;13:21544. doi: 10.1038/s41598-023-48435-4 (PMC10700580; doi:10.1038/s41598-023-48435-4)
Supplement: Supplementary file 1 — Supplementary Information. [file 41598_2023_48435_MOESM1_ESM.docx]

**SUPPLEMENTARY**

**Supplementary Table** **1:** miRNAs analysis. Untreated HCM1 cells exosomes and H1299 membrane activated HCM1 cells exosomes represent the average of 2 independent experiments in the same sequencing assay.

| miRNA | Untreated | Memb Activated | BaseMean | log2(FC) | stdErr | Wald-Stat | P-value | P-adj |
| --- | --- | --- | --- | --- | --- | --- | --- | --- |
| miR-31-5p | 16.77 | 1702.91 | 859.84 | -4.02 | 0.50 | -8.12 | 4.85E-16 | 1.02E-12 |
| miR-100-5p | 217.42 | 2622.23 | 1419.83 | -2.43 | 0.49 | -5.01 | 5.42E-07 | 0.000568 |
| miR-125b-5p | 358.56 | 3054.62 | 1706.59 | -2.26 | 0.46 | -4.90 | 9.73E-07 | 0.00068 |
| miR-99a-5p | 199.99 | 1106.10 | 653.05 | -1.77 | 0.47 | -3.77 | 0.000166 | 0.086854 |
| miR-137 | 2.34 | 29.56 | 15.95 | -1.91 | 0.52 | -3.66 | 0.000251 | 0.105031 |
| miR-193a-3p | 75.37 | 390.41 | 232.89 | -1.65 | 0.48 | -3.43 | 0.000601 | 0.209977 |
| miR-6771-5p | 263.16 | 1653.48 | 958.32 | -1.63 | 0.51 | -3.22 | 0.001301 | 0.389488 |
| miR-181d-5p | 21.58 | 106.10 | 63.84 | -1.54 | 0.49 | -3.16 | 0.001565 | 0.41004 |
| miR-196a-5p | 36.44 | 159.13 | 97.79 | -1.41 | 0.49 | -2.87 | 0.004048 | 0.925361 |
| miR-4324 | 9.34 | 44.61 | 26.98 | -1.43 | 0.51 | -2.82 | 0.004856 | 0.925361 |

**Supplementary Table 2:** Genes regulated by the two miRNAs.

| **Target genes** | **Total** | **miRNA** |
| --- | --- | --- |
| AKT1 ATP2A2 BMPR2 CAPNS1 CREBBP EEF1A1 HMGN1 IGF1R IGF2 SMAD7 MMP13 ORC5 PAFAH1B1 PCBP2 SET PTP4A1 CUL1 SMARCA5 SNX4 QKI NCOR2 MORF4L2 EDEM1 TRIB1 ZBTB7A GDE1 QSER1 KLHL15 SOGA1 PIKFYVE UBN2 LIN28B CFLAR CD4 PTBP1 HMGB3 PIK3CB FNDC3B BAZ2A EDN1 KHSRP TNRC6B EIF5 YY1 ACIN1 XIAP RAB2A EEF1D HOXA3 TSPAN14 SLC16A6 HIPK3 GLI1 PPM1H CDKN1A SMO INO80 RBM39 NUP210 BTBD3 THBS1 SLC39A6 PARP1 COPS7B UHMK1 RMND5A SORBS2 FAM126B SLITRK5 C18orf54 ATXN2L GFM1 FASN SIK2 BCL2 ZNF483 MGA ZBTB34 SOX12 POU3F2 INTS5 | 81 | miR-100-5p, miR-125b |
| ACTB AKT1 ALDH9A1 APEX1 ATM ATP1A2 ATP1B3 ATP2A2 ATP5A1 BMPR2 CAPNS1 CCNG1 COL4A1 CREBBP DNMT1 EEF1A1 EGR2 EIF5A ESR1 EWSR1 ACSL3 FGFR3 FKBP5 FLT1 FXN MTOR XRCC6 GNG5 GRB2 GTF2H1 H3F3A HMGB1 HMGN1 HOXA1 FOXN2 IARS ID1 IFIT3 IGF1R IGF2 CYR61 INSIG1 KPNA2 LDHB ABLIM1 SMAD7 MAFG MAP4 MKLN1 MMP13 COX2 CYTB NDUFA2 NDUFA9 NDUFC2 RPL10A NFIA NOP2 NSF ODC1 ORC2 ORC5 OXA1L OXCT1 PAFAH1B1 PRDX1 PCBP1 PCBP2 PFN2 PLK1 PPP1CB MAPK6 PSMA2 PSMA5 PTPN9 RAB5C RAD51C RAP1B RARS RB1 RCN2 RPL5 RPL7 RPL7A RPL10 RPL15 RPL19 RPL21 RPL26 RPL31 RPL36A RPLP1 RPS8 RPS15 RPS15A RPS27 RRM2 S100A10 SAT1 SET TAPBP TCP1 UBB USP1 VCP XRCC5 YWHAE ZNF215 PTP4A1 CUL5 HIST2H2AA3 HIST1H4C CUL2 CUL1 SMARCA5 YBX3 BECN1 SNX4 TSC22D1 BCL10 BAZ1B RPL14 PAPSS1 DDX21 COX5A QKI ITM2B GOSR1 APBA3 H2AFY GTPBP1 NCOR2 MORF4L2 EDEM1 DAZAP2 HS3ST2 MED12 UBA2 FARSB ACTR1B ALG3 CTDSPL TRIB1 UBE2E3 LRRC41 DEAF1 ATP5L CAMKK2 GNA13 ARPP19 WDR4 PNRC1 KDELR2 SNRNP27 VPS45 CBX3 DOLK SMG1 KANK1 PRRC2C SMCHD1 SF3B3 DDAH1 PATZ1 MTO1 UNC50 SERBP1 AP3M1 FOXP1 SMPDL3B GRHL1 UBQLN1 PRELID3B SEPSECS RLIM HSPA14 ZBTB7A TEX264 NIP7 HACD3 CTDSPL2 ANAPC11 MBIP GDE1 MRPS33 C21orf91 TERF2IP GFOD1 SGTB NDE1 MAP7D1 N4BP2 TMEM30A RIOK2 DCAF6 JPH1 TM9SF3 AGPAT3 PSMG2 ACKR3 RAP2C ANAPC1 MARC1 FBXL15 CDC73 SRD5A3 PANK3 SAP30L KATNBL1 QSER1 ATAT1 CTC1 KLHL15 RAB33B THAP2 KBTBD8 UTP4 RHPN2 YTHDC1 ATP6AP1L MAPK1IP1L ORMDL1 H2AFV NLRP3 RPL39L ZNF511 NTAN1 WTIP LIX1L MITD1 NUDCD2 UBXN2B SOGA1 EIF5AL1 SEPT10 CCNYL1 C9orf66 PIKFYVE CCZ1B TMED4 ZNRF2 SLC17A8 UBN2 RNF144B SREK1IP1 DCBLD1 RPL7L1 DPY19L4 FAM221A LIN28B HIST2H4B BMPR2 DNMT1 FGFR3 FKBP5 FXN MTOR GRB2 HMGN1 HOXA1 IGF1R MAP4 MMP13 NDUFC2 NFIA NSF ORC2 PRDX1 PCBP1 PCBP2 RPL36A RPLP1 UBB VCP PTP4A1 CUL1 SMARCA5 SNX4 COX5A DAZAP2 HS3ST2 UBA2 CTDSPL ARPP19 PNRC1 SF3B3 DDAH1 UNC50 SERBP1 FOXP1 GRHL1 UBQLN1 HSPA14 CTDSPL2 MBIP GFOD1 TMEM30A RIOK2 DCAF6 AGPAT3 ANAPC1 KBTBD8 ORMDL1 ZNRF2 RNF144B DPY19L4 DPM1 CFLAR TFPI CD4 PTBP1 CLDN11 NPC1L1 HMGB3 RFC1 GOPC PIK3CB ALX4 ROCK1 NEDD4 SLC44A1 DGCR2 ASNS ATP2B1 ATP6AP1 TRIB2 SEMA3C FNDC3B FSCN1 BAZ2A GTF3C1 MAP2 EDN1 CBFA2T2 RIMS1 MEF2C MRPL22 FOLH1 EPDR1 NID2 KHSRP GNA11 GOLGA3 CD209 SLC7A8 NANS CIRBP RANBP1 CYB5R3 TNRC6B RANGAP1 EIF5 ZC3H14 YY1 ACIN1 DNAJC5 MID1 XIAP CD99L2 EEA1 MGRN1 RAB2A EEF1D HNRNPL CLPTM1 OLFM2 SIGLEC6 CDK6 HOXA3 NUDT1 PTPRZ1 IMPDH1 LSM5 GLCCI1 CPEB3 SMC3 TSPAN14 CYP2C18 LGALS3BP CCL7 CCL2 SLC16A6 INPP4B KLF3 ZBTB16 SIAE HIPK3 KCTD10 GLI1 PPM1H SUDS3 RNF8 PERP HECA GMDS TTK NPR3 CPEB4 CEP70 KLHL24 HSPE1 ABCB6 STK25 CEP104 DLEU2L SLAMF1 HSD11B1 KIF14 CCDC92 GPAM NFYB DCAF10 CISD1 ATPAF1 LPGAT1 CTCFL CDKN1A CD93 SGPP1 EVI2A SMO INO80 KIF1C CDO1 PHF10 NXNL2 ACE2 TULP4 SNX9 SESN2 CASZ1 RBM39 PSMC3IP NUP210 RAMP1 BTBD3 TPT1 TMTC1 CCDC59 ARL8B TMEM165 ADAM19 DYSF RCBTB1 IGF2BP3 GPNMB RAC1 IL6 CIB2 NMT1 DENND4C FDX1 THBS1 TUBGCP4 DNASE2B SSB AP1AR CNOT6L PPP3CA ANKRD52 RAB15 ARRDC4 RPS2 SLC39A6 CACNG8 SF3B4 PIP5K1A DEGS1 PARP1 TMEFF2 COPS7B GOLGA4 PHLDB2 CAMK2D SLC2A12 PURB VLDLR STOM HABP2 PAMR1 SESN3 C11orf52 SLC7A11 GXYLT1 CACUL1 KCNE4 SPC25 PDK1 UHMK1 CENPH SCN3A RMND5A CCSAP SORBS2 ATP6V1C1 C16orf87 FAM126B FZD7 CXCL13 WDYHV1 TAGLN2 NAE1 SRSF2 CXCL16 SLC25A45 DNAJB4 FRZB FZD5 GMPS SGMS2 HSPA4L C4orf3 MAP9 CASP3 FABP5 CCDC171 GKAP1 CLDN3 SLITRK5 REEP3 BMS1 SERPINA12 TRMT61A ARIH1 TMEM41B TMEM135 MMP10 C18orf54 NKX3-1 SGSM1 TBC1D16 RHEBL1 CHAF1A RBPJ KCTD6 ATXN2L DYNLRB2 GFM1 FEM1B PCSK9 FASN CHD3 GPR37L1 SIK2 HSPB2 B3GNT2 SLC30A1 CD14 FUT3 CLCN5 KCND3 HOPX PPID CAMTA1 GATM BCL2 ZNF217 SERF1A ISG20 BPGM MRPL57 ZNF483 PSMD1 TOPAZ1 PHC3 MGA PDE12 RSRC1 PLEKHF2 DENND2C LMNB2 TYMS ZBTB34 FZD8 THAP5 SOX12 GRAMD1C GALNT11 WDR6 ZNF852 OR13D1 PTPN11 GREM2 HIGD1A TMEM132E ZNF322 BGN PAPPA TSPYL2 POU3F2 FLRT2 INTS5 POU3F1 MYBL1 RASA3 CYP4F2 RGS9BP NAP1L1 MAGI2 KCNJ11 RNFT1 PPIA CD47 SPRR2B LRRC8B TTC30A TAF13 FAM49A NKIRAS1 RPL12 AKR1B10 HMGN5 HYLS1 EIF1AY SPRR2E ARRDC1-AS1 RPA4 MAFB RUFY2 ZDHHC18 OR14J1 SERF1B FAM99A DIO1 TRGC1 MYCBP PLCXD2 HOXA10 RBM15B CCL5 PCDHB16 ARHGAP23 | 577 | miR-100-5p |
| ABL1 ACLY ADM PARP1 AKT1 ALOX5 ANGPT2 APC XIAP ARF3 ATP5B BACH1 BAK1 BCL2 BCL2L2 BCL3 PRDM1 BMPR1B VPS51 CBFB CCNE1 SCARB2 CD44 CDH5 CDKN2A CDKN2D CEBPA CEBPG CNGB1 COX7C CREBBP MAPK14 CSNK2A1 CYP24A1 ARID3A DUSP6 E2F2 E2F3 EEF1A1 MEGF9 EGFR EIF4E EIF4EBP1 EIF5 EMD ENPEP EPHA7 EPO EPOR ERBB2 ERBB3 ETF1 ETS1 EXTL3 FAT1 FBN1 FES FGFR2 FUS GABRB3 GLI1 GOLGA1 MKNK2 GRIN2A GRINA GSS HDGF HK2 HMGN1 HMGA1 HNRNPK HOXA13 HOXD1 HSPA1B HSPD1 ICAM2 IGF1R IGF2 IL6R IRF4 JAK2 KCNS3 KPNB1 KRTAP5-9 KRT7 LIF LIFR LIPA LSS LTA4H MXD1 SMAD4 MCL1 MAP3K11 MMP2 MMP13 ABCC1 MTF1 ND6 MUC1 NEU1 NF2 NME2 NPM1 NRDC NTRK3 OPRL1 PAM PCBP2 PFKM PIGF PIK3CB PIK3CD PKM PMAIP1 PODXL MED1 PPAT PPP1CA PPP2CA PRKAG1 PSMB1 PTBP1 PTGS1 PTH1R RAB4A RAF1 RANBP2 REST RNH1 RPL3 RPL29 RPL35A RPLP0 RPS3A RPS6KA1 RPS7 RPS12 RPS28 SORT1 S100A8 ATXN1 SCNN1A SDHB SEL1L SET SFRP5 SHMT1 SIX1 SLC7A1 SLC19A1 SLC25A1 SMARCD2 SMO SNRPB SSR3 STAT3 SUPT6H SUV39H1 TDG PRDX2 TEF TNF TNFAIP3 TP53 UNG VCL VDAC1 VDR ZNF177 BTG2 IFRD2 ZNF212 KMT2D FXR1 HMGA2 TAF15 FZD4 FZD6 DYRK2 IRS4 KHSRP PRKRA STC2 DGAT1 B4GALT3 PABPC4 TNFRSF10B APLN SPHK1 SGPL1 MTMR3 SLC7A6 CLDN12 USP8 MTMR4 SLC16A4 AURKB MFHAS1 SLC9A3R2 RASAL2 VPS4B TMEM59 PTGES NCOR2 ZNF592 NUP93 EDEM1 STARD8 RNF144A MATR3 DHX38 IP6K1 KIAA0141 GAB2 CEP170 UBAP2L THRAP3 HUWE1 RASGRP1 SUGP2 ABCC4 RGS19 BCKDK SMYD5 PIAS3 ARIH2 C1D SEMA4B NEBL RNASEH2A TACC2 SMC2 ARID3B SPHAR MAP3K2 NES HBS1L ZMYND11 SEC24A EHD1 ILVBL ADRM1 AKAP2 MAN1B1 LYPLA2 FAS CASC3 IKZF3 IKZF2 COPZ1 R3HDM2 MON1B BTBD3 SPEN GOLGA8A EMC1 TNRC6B PLXND1 PHF8 NUP205 GANAB TBC1D1 SIK2 PDS5A EXOC7 FAM208A CRTC1 FRAT2 RYBP TARDBP SRRM2 NNT TNPO3 CARHSP1 HSPBP1 TMEM2 BCL2L13 LSM4 BACE2 THUMPD3 ZDHHC5 ZNF385A ULK3 TIMM10 PABPC1 NPTN BBC3 DKK3 NDOR1 RRP7A TOR2A NKIRAS2 STRN3 PSAT1 MYEF2 MRTO4 PDZD11 CD320 SIRT7 KLF13 CHMP3 CD244 LUC7L3 ZFYVE1 PANK1 PTOV1 CHPF2 HMGCLL1 MTRF1L MTMR12 CCNJ TET2 BCOR NSUN2 SEMA4C C1orf109 C17orf80 UBR7 RFWD3 LINS1 UBA6 YOD1 THUMPD1 NDC1 WDR11 ZNF395 PCDHGB2 MEPCE MMP26 EIF5A2 C15orf39 DHX33 STOX2 PLXDC1 SMIM8 TMEM63C PLEKHG5 PPM1H AHRR CGN ZSWIM6 CTDSP1 PCTP SINHCAF HRH4 IKZF4 UNKL IPPK RMND5A KLC2 WNK1 DDX50 NUP37 RSG1 DYNC2H1 SMC6 LIN28A EHMT1 PIP4K2C ESRP2 FBXL18 MYO19 ABTB1 SH3BP5L FBXO38 CSRNP2 SPRTN C2orf88 EIF1AD TMEM101 ACSS1 PLEKHA8 MFSD9 MUM1 LTV1 FBXL20 TNKS1BP1 LBX2 THAP3 BMF STARD13 DHX57 FMNL3 ASB16 TP53INP1 SLC35A4 LACTB GPRIN1 CCDC124 ANAPC16 DRAM2 TRIM71 GPAT4 FAM199X SAMD10 RIMS4 E2F7 CMTM4 LSM14B C2orf15 FAM91A1 ZNF483 SLC35G1 LONRF2 SPATA5 QSOX2 RHOV NBPF11 TMEM136 TMEM9 PLA2G4F NEGR1 SEC14L3 HID1 CDRT4 C19orf54 PPP1R37 HKR1 MMAB KIF24 GALNT18 GJB7 LIN28B FAM174B CENPP GOLGA8B HIST2H2BF NRARP ANKRD33B TIMM23 HOTTIP KLF1 PARP1 APC XIAP BAK1 BCL3 SCARB2 CEBPG CSNK2A1 ARID3A DUSP6 E2F3 EEF1A1 MEGF9 EIF5 EMD ENPEP EPO EPOR ERBB2 ERBB3 FGFR2 FUS MKNK2 GRIN2A GSS HDGF HK2 IGF1R IGF2 IRF4 LIFR LIPA MCL1 MUC1 NME2 NTRK3 PKM PMAIP1 MED1 PPP1CA PPP2CA PSMB1 REST RPS6KA1 RPS12 RPS28 SORT1 ATXN1 SCNN1A SEL1L SET SLC7A1 SMARCD2 SMO STAT3 SUV39H1 TDG TP53 BTG2 IFRD2 KMT2D HMGA2 DYRK2 IRS4 PRKRA STC2 PABPC4 TNFRSF10B SGPL1 AURKB MFHAS1 VPS4B NCOR2 STARD8 UBAP2L SUGP2 BCKDK SEMA4B TACC2 ARID3B EHD1 LYPLA2 IKZF3 IKZF2 COPZ1 MON1B GOLGA8A TNRC6B NUP205 GANAB PDS5A EXOC7 SRRM2 NNT BCL2L13 LSM4 ZNF385A PABPC1 BBC3 TOR2A PDZD11 KLF13 ZFYVE1 CHPF2 CCNJ TET2 SEMA4C YOD1 THUMPD1 ZNF395 C15orf39 DHX33 STOX2 ZSWIM6 CTDSP1 PCTP IKZF4 KLC2 WNK1 NUP37 DYNC2H1 LIN28A SPRTN EIF1AD TMEM101 ACSS1 MFSD9 FBXL20 TNKS1BP1 STARD13 FMNL3 TP53INP1 SLC35A4 LACTB GPRIN1 ANAPC16 FAM199X LSM14B ZNF483 SLC35G1 RHOV PLA2G4F NEGR1 PPP1R37 LIN28B KLF1 ANKIB1 CFLAR CDC27 GDE1 TNFRSF12A KDM7A COX10 GTF2IRD1 ADIPOR2 PAFAH1B1 SELE JARID2 SPAG9 PKD1 CSDE1 UBE3C NCAPD2 CD4 HIVEP2 ELOVL5 XYLT2 RUFY3 AGPS NLRP2 AKAP11 UBR2 KPNA6 TNFRSF1B SLC39A9 HMGB3 GRN VCAN CTNNA1 DSG2 TPR FAM120A MCUR1 FAM160A2 HERPUD1 KIF1B SZRD1 CUL1 CYFIP2 USP36 KMT2C RC3H2 MTA3 ATP11B ZC3H11A TBXAS1 PARP12 QSER1 BCAT1 BTBD1 MYLK SEC61A2 CDK13 MTHFD2 FOXJ2 DNTTIP2 SDK2 NUP133 NUCKS1 RORA KIAA1107 MNT MBD3 TFRC MPP5 NFATC3 EVC MAP3K13 PICALM CA12 FNDC3B BAZ2A NT5C2 CAMSAP3 DGKD PAK3 FGFR1 PPP2R5C EDN1 TNRC6C SENP1 PGR CCNT2 NFE2L1 GSK3B XPO1 STARD7 TXLNA CD59 SCAMP1 EPS15 B4GALT1 TMED2 ALG9 MTMR2 SH3BP2 ADD1 TPX2 NSFL1C SNX5 PXN PEBP1 CDIP1 ICAM1 TNRC6A OSBPL8 PUS7 CCDC80 EZR AGO1 GPATCH2 SEC22C CDC6 ARCN1 WAC BRPF3 SCD NRP1 TSPAN15 FBXL19 TECR TIMM13 CRKL DDX17 AP1B1 MYH9 FAM118A ZC3H7B KHNYN NIN DAAM1 PPM1A CEP128 DICER1 YY1 ACIN1 PABPN1 CHD8 ZMYND8 ADNP NELFCD SEC23B MYL9 SMAD7 ANKRD12 STS JADE3 CDK16 FGF14 NDFIP2 POLR2C AXIN1 CAPN15 MAZ RAB11A TMEM87A ZNF106 ZFAND1 NBN UBE2W POP1 RAB2A NDRG1 EEF1D SLC39A14 R3HCC1 SLC17A7 OAZ1 TIMM44 PLD3 SLC1A5 HNRNPUL1 KDELR1 TNPO2 ISYNA1 SIPA1L3 TWISTNB HBP1 HOXA3 LFNG GRB10 COBL TAF6 SERPINE1 TMEM248 TBC1D13 NPDC1 RAB11FIP2 SEC23IP PLEKHA1 LZTS2 PPIF TSPAN14 MED13 RNF167 DDX5 PPP1R9B SLC16A6 PRKAR1A CDR2L ELF2 TRIM2 GLRB RAPGEF2 HTATIP2 VWA5A EIF4G2 DDX6 HIPK3 CD81 PRDM4 CORO1C SLC11A2 MLEC PTGES3 ITFG2 FOXM1 CDKN1B SLC38A1 CPSF6 CDCA3 BTN3A3 TMEM14C MAN1A1 SERINC1 ULBP1 SOD2 PTP4A1 SIM1 QKI SRF VEGFA SPARC HMGCR TTC1 LMNB1 ARRDC3 NUP155 SNX4 EIF1B MOB1A TTL PIKFYVE NCL ACTR3 ACVR1 FNDC4 PSMD14 SNX17 NDUFS7 RTN4 LANCL1 EFEMP1 GLS USP34 EFHD1 GGCX PDCL3 BIRC6 STRN SLC25A12 COX7A2L TRAK2 EPAS1 NFE2L2 EPHA4 BCL9 PRRX1 FAM20B CHD5 STXBP3 EDEM3 CAPZA1 RNF19B GON4L SRSF11 MTR ETV3 PTCH2 BLZF1 RSRP1 NSL1 ARID1A CENPF RPA2 STK11 CAMSAP2 CREB1 ELOVL4 PLAGL1 MFSD1 PPL UBN1 UCHL3 HS1BP3 GTF3C3 SATB2 RAB14 MAPKAP1 AREL1 ALDH6A1 HELLS HOXB5 CAAP1 WDR55 PROSER1 ELF1 KBTBD7 DUSP4 LYPLA1 AKAP1 DESI2 LRIF1 GPSM2 ZC3H13 NR4A1 AMD1 MORF4L2 AGO2 FAM210B NCOA3 VAPB PARD6B ARFGEF2 TMEM189-UBE2V1 STX16 SIRT5 CDKN1A SOX4 RREB1 SSR1 AHNAK CNOT1 EFNB2 SLC25A19 PPP1R12C CCDC93 STK35 BMP2 MAX DYNLRB1 PLAGL2 AGO3 TUBGCP3 CAPNS1 THRA BCL2L12 PLEKHG3 EPS15L1 MACF1 MTERF1 CALU KLHDC10 NDUFA5 MYO1B INO80 CHAC1 CLN6 VPS13C MPDU1 PHF20L1 MTUS1 SH3BP4 LDLR LRCH2 KIF1A MRPL34 RSPH3 SULT4A1 POMT1 EIF2S3 LRP3 SYNE1 RBM39 GSE1 SLC6A6 KIF3A DIAPH1 MAP1B ZSWIM4 NUP210 RRP8 PER2 RPA1 GRSF1 UNK SERINC3 DSTYK DCLK1 RTN3 ZFC3H1 LOXL2 IER3IP1 BHLHE40 SLC38A2 YWHAQ KIDINS220 ROCK2 IAH1 IL6ST PHC2 CDCA8 CLOCK MDFIC PRRG4 CAPRIN1 DNAJC14 CCDC142 DYNC1LI2 STX6 DHX9 CEP350 RC3H1 VPS36 COG3 RAPGEF5 DGKB ALPK3 VEZF1 SCN2A TBR1 LRRC8A ATP6V1G1 DPM2 DNAJB5 ANKRD42 NUMA1 SORL1 YAP1 PPP2R1B THBS1 ZNF280D ARHGAP29 PPM1B ACTR2 TET1 TMOD3 HNRNPD KIAA1109 G3BP2 SEC24B FBN2 GABARAPL1 FGD4 PRICKLE1 SCAF11 PPFIA2 TMBIM6 SBNO1 VPS37B ZSCAN29 NCOA2 DNAJA4 POLG FURIN IQGAP1 ZFHX3 CDH11 MBTPS1 CDH13 GFOD2 NPEPPS SSH2 CLTC SS18 SLC39A6 MINK1 TRIM65 IGFBP4 BRD4 APP SAE1 LMTK3 CARM1 IGSF3 ATP1B1 POGK POU2F1 ABL2 C1orf43 GATAD2B RIT1 FLG SDE2 FBXO28 ARF1 CDC42BPA MBOAT2 PLEKHA6 TMEM177 COPS7B CSRNP1 TMF1 LRIG1 NXPE3 SLIT2 ANK2 G3BP1 LRRTM2 PHIP MMS22L IGFBP3 GIGYF1 TRIM4 OGT DOCK11 ATP6V1B2 MTDH CDKN2B FBXO10 SIGMAR1 NTRK2 UGCG NR6A1 MED22 GTF3C5 GPR107 EIF4EBP2 MKI67 LIN7C NCAM1 TMEM138 SOGA1 DLG2 CRIM1 ANKRD50 ADAM17 SACS PABPC3 DST PTPN14 EPG5 TGOLN2 UHMK1 ZNF776 GPR180 SMARCA5 RASSF3 HNRNPU PTPRD CHD1 THY1 PRKCA SORBS2 ADAMTS5 SLFN13 LARP1 FAM126B SLC24A2 RASA2 PSD3 ELMSAN1 TIAM1 SUPV3L1 PRR14 BUB1B BRPF1 FCHO2 SYN2 APPL1 UBN2 PSMG3 XKR8 WASF2 ABHD3 CDC25C EIF5B CNOT11 NBL1 DEDD ADAMTS4 CACHD1 SON SV2A IGF2BP1 SIM2 ALDH4A1 AMFR EFCAB14 ZNF362 RRP1 C21orf58 ZNF714 TAOK1 PCSK7 ADAR NBEAL2 IER2 RPL8 LARP4 TTC9C SYVN1 TPCN2 CMPK1 KLHL21 RAVER2 KIAA1522 ZNF281 PEA15 CNST MAPKAPK2 WDR26 PEX13 KCNJ3 IVL LRRC58 RPP14 SNRK LZTFL1 CDC25A SHISA5 NIPBL ACSL6 TMEM200A BMPER ZNF12 ZNF704 DLC1 ORC5 SUN1 ZHX1 DYNLT3 SLITRK5 PCF11 ARF6 AMER2 MOAP1 CACNB2 SPRED1 HIF1AN CDYL2 SLFN5 ANPEP C18orf54 STX3 YWHAB MAPRE2 FAM102A URM1 CRK CDK12 SERTAD3 LENG8 EEF2 KRT80 OTUB1 VPS37C SLC3A2 CTNNB1 NUDT16L1 KIF5C PDHB ATXN2L GDNF AXIN2 STX18 GFM1 LETM1 SIN3A CLIC4 FASN DUS1L UGP2 HNRNPF PCDH7 WNT10B ZNF768 ZNF35 YWHAG HNRNPA3 PWWP2A CRTAP DENND5B KIF5B CDCA4 PPM1D TRIAP1 KIAA0232 PDGFD INSR MFN1 TMEM126A NETO2 NPTX1 CHD7 ZNF562 MAP6 SLC25A33 CTPS1 BSG POP7 GNB2 C2CD2L GTPBP2 MANEA DCP2 LCLAT1 TADA2B FAM222B KDM2A GOLGB1 TRIB1 SMARCC1 CSPG4 RCE1 HEG1 CNP RNF213 SWSAP1 KLHL15 FBXO45 MGA ATP2A2 CNTNAP2 GCSAM AKIRIN1 ASPHD1 ATR VCPIP1 ZNF654 PTPN2 KCMF1 B3GNT5 BASP1 NFATC2IP SEC24C SLC38A9 ZBTB34 RIMKLA PUS1 CHD9 KCNA2 ZBTB38 ST8SIA3 JUN SOX12 PDE4DIP SH2B1 ZNF543 PLEKHM3 GLDC SUZ12 NSUN3 ZBTB7A EIF3K SNX18 EDC3 PDXDC1 PAK2 NRIP1 GPR157 ZNF792 CUEDC1 TCEAL8 AEN MUC16 RAP2B TNFSF15 YIPF6 IBA57 GINS3 TMEM259 NOP10 IST1 CREB3L2 FIGN MFSD5 COL18A1 SPATA13 AFMID PRR14L TMEM50A KCTD16 ZNF703 RBM12B TOB2 ACTG1 C22orf46 IRAK1 PCDH9 ALDH1A3 POU3F2 PIWIL3 ATL3 ARMCX2 INTS5 ZBTB37 RAD51D LSAMP SP1 P4HB PBX1 BRWD1 YTHDF3 DMWD LAMP1 POMK KRT5 PIP5K1C KIF18B PPP1CC KRT10 KPNA4 HEXIM1 PPARA TEAD1 GCNT1 TET3 SEMA4D MCRS1 PTAR1 TMEM201 FAM120AOS ZNF292 KCTD21 ARID2 TSPYL1 FAM53B FAT4 TMEM63A WDR5 ARMCX4 YRDC ZNF775 AFAP1 RABL6 ZFP62 ARHGEF12 FLNA WDR45 ZNF398 MIRLET7BHG NOL4L DDI2 ADARB1 SIPA1L1 SPTAN1 ZNF460 PSAP ZNF121 VPS13A ZBTB14 CD2AP QRICH1 DDX42 GFPT1 TXNRD1 YTHDF2 ECI2 UNC13B SLC5A3 PLXNB3 SFT2D1 CHAMP1 RBM20 ZDBF2 BMPR2 EHMT2 PRRC2A ZNF805 DDR1 1 GABBR1 ZBTB48 FBXO48 RNPS1 SFT2D2 ZNF254 NRAS LEPROT S1PR3 NUDT19 REPIN1 HOMEZ DDX3X PSG7 SMIM13 ERCC6 BOD1L2 RBM14 NME1 PCDHGC3 PNMA2 PISD UGT1A1 EIF6 PPAN-P2RY11 TMED7-TICAM2 C8orf88 ZNF260 PCDHGA4 DYNLL2 TXNIP RASSF5 NCOA4 RASL10B NUDT3 KMT2B MLLT6 PIP4K2B PIGW ZNF8 | 1338 | miR-125b |

**Supplementary Table 3:** CircRNAs regulated by the two miRNAs.

| **Target circRNAs** | **Total** | **miRNA** |
| --- | --- | --- |
| MTOR E2F2 ARID1A TMEM87B RGPD6 RGPD8 ANAPC1 TTN CAND2 CTDSPL LPP FGFR3 EVC VCAN UHRF1BP1 LIN28B SUN1 POM121C HIP1 PAXIP1 ASAP1 DENND4C ANKRD18A SLC44A1 DENND1A RAPGEF1 ZDHHC5 FAM111A IGHMBP2 SORL1 WNK1 MLF2 SCAF11 SLC38A1 DIP2B GOLGA3 STAT5B ITGB4 RPTOR PIK3R2 C19orf54 MTMR3 TNRC6B GNL3L | 44 | miR-100-5p, miR-125b |
| ICMT MTOR E2F2 PAFAH2 ARID1A LRRC41 ATPAF1 PIGK RAP1A SF3B4 IQGAP3 DTL TRIB2 NCOA1 DYSF RNF149 TMEM87B RGPD6 RGPD8 ANAPC1 PDK1 TTN PSMD1 CAND2 SLC4A7 CTDSPL WDR48 SEC22C SNRK CDC25A KBTBD8 ZBTB20 PLXND1 MBNL1 LPP RPL35A FGFR3 HTT EVC PCDH7 PPP3CA VCAN CCNH RIOK2 GMDS UHRF1BP1 LIN28B SUN1 PPIA POM121C HIP1 AASS CNOT4 EZH2 SMARCD3 PAXIP1 VCPIP1 STK3 ATP6V1C1 ASAP1 DENND4C ANKRD18A SLC44A1 DENND1A ST6GALNAC4 RAPGEF1 GDI2 TTC17 ZDHHC5 FAM111A RBM4B PC IGHMBP2 LOC100133315 VPS11 SORL1 WNK1 MLF2 ITPR2 SCAF11 SLC38A1 DIP2B SCN8A THAP2 NDUFA12 NT5DC3 GOLGA3 RCBTB1 TUBGCP3 HNRNPC SALL2 HECTD1 ZC3H14 RPS6KA5 MYO9A IREB2 RPUSD1 RRN3 ZNF19 WDR59 CXCL16 ACADVL HS3ST3B1 TAOK1 ACLY STAT5B FTSJ3 ITGB4 CANT1 RPTOR SMAD4 TSHZ1 CIRBP MBD3 THOP1 UBXN6 PIK3R2 TBCB ZFP14 HNRNPL C19orf54 VRK3 SALL4 ZNF217 MTMR3 TNRC6B TBL1X GNL3L OGT ATP11C | 130 | miR-100-5p |
| AGRN MXRA8 ATAD3C ATAD3A SLC35E2B NADK PEX10 LRRC47 GPR153 NOL9 H6PD SPSB1 UBE4B KIF1B PEX14 AGTRAP DHRS3 DDI2 SPEN EPHA2 CROCC MFAP2 ARHGEF10L MUL1 EIF4G3 ECE1 HSPG2 LUZP1 HNRNPR ASAP3 LYPLA2 TMEM50A LDLRAP1 ZNF593 CEP85 ZDHHC18 NUDC WDTC1 WASF2 PTAFR YTHDF2 KPNA6 HDAC1 ZNF362 PHC2 ZMYM6 SFPQ ZMYM4 KIAA0319L MAP7D1 CDCA8 MACF1 PPT1 RLF RIMS3 ELOVL1 PTPRF DPH2 HECTD3 NASP MAST2 NSUN4 STIL CC2D1B ZYG11A USP24 FGGY RAVER2 LEPROT ANKRD13C ZZZ3 PKN2 TGFBR3 DNTTIP2 AHCYL1 CAPZA1 MOV10 RHOC PHTF1 NRAS CSDE1 PTGFRN TTF2 NOTCH2 NBPF9 NBPF10 LOC653513 PDE4DIP TXNIP BCL9 NBPF15 PRPF3 MCL1 CDC42SE1 ZNF687 SNX27 S100A14 CHTOP UBAP2L HAX1 UBE2Q1 PBXIP1 GON4L YY1AP1 SSR2 RAB25 LMNA SMG5 TMEM79 MEF2D NES B4GALT3 UCK2 BLZF1 F5 PRRC2C PRDX6 RC3H1 RASAL2 CEP350 LAMC1 RGL1 TPR PKP1 PPP1R12B KDM5B BTG2 ZC3H11A DSTYK MAPKAPK2 HHAT PTPN14 CENPF LYPLAL1 RAB3GAP2 MIA3 FBXO28 TMEM63A PARP1 PSEN2 TAF5L C1orf198 CHRM3 HNRNPU ZNF124 PXDN RPS7 ASAP2 RRM2 HPCAL1 NBAS APOB ATAD2B AGBL5 PREB SLC5A6 FNDC4 LBH LCLAT1 BIRC6 CRIM1 PPM1B SRBD1 PRKCE EPAS1 PSME4 SPTBN1 PNPT1 EFEMP1 XPO1 UGP2 VPS54 AFTPH RAB1A PCYOX1 ZNF638 RAB11FIP5 TET3 DCTN1 SLC4A5 CCDC142 MRPL53 AUP1 GGCX VAMP5 PTCD3 SEMA4C ACTR1B INPP4A C2orf15 MRPL30 NPAS2 MAP4K4 EDAR RANBP2 RGPD4 SH3RF3 LIMS1 MALL ZC3H6 ERCC3 AMMECR1L UGGT1 PTPN18 NCKAP5 MGAT5 CXCR4 LYPD6 RIF1 PKP4 TANC1 BAZ2B UBR3 CDCA7 AGPS CCDC141 NUP35 COL3A1 COL5A2 GLS SLC39A10 SPATS2L CFLAR INO80D METTL21A PIKFYVE ATIC FN1 MREG CTDSP1 USP37 STK36 TTLL4 COPS7B DIS3L2 EIF4E2 GIGYF2 DGKD ARL4C AGAP1 COL6A3 UBE2F-SCLY RNPEPL1 PASK D2HGDH SUMF1 ITPR1 IRAK2 TATDN2 SEC13 ATG7 VGLL4 SYN2 RAF1 SLC6A6 EAF1 BTD RFTN1 TOP2B TGFBR2 CMTM7 CMTM6 GORASP1 VIPR1 LZTFL1 SCAP DHX30 MAP4 SHISA5 NCKIPSD PRKAR2A QRICH1 IP6K1 UBA7 GNAI2 HYAL1 RAD54L2 TEX264 TWF2 NISCH STAB1 ITIH3 SFMBT1 ACTR8 FLNB PTPRG ATXN7 PSMD6 LRIG1 SPICE1 GOLGB1 ITGB5 HEG1 SLC12A8 SLC41A3 TXNRD3 PLXNA1 ABTB1 NPHP3-ACAD11 CDV3 DBR1 RASA2 GMPS RSRC1 FNDC3B TBL1XR1 ATP11B MCCC1 KLHL24 DVL3 ALG3 ST6GAL1 XXYLT1 SDHAP2 RNF168 LETM1 ZFYVE28 TNIP2 RGS12 KIAA0232 TBC1D14 TADA2B AFAP1 CLNK NCAPG LCORL PDS5A ATP10D FRYL FIP1L1 PPAT GRSF1 ANKRD17 USO1 SCARB2 SHROOM3 ENOPH1 SEC31A WDFY3 AFF1 SLC39A8 TET2 SEC24B ELOVL6 KIAA1109 SPATA5 ANKRD50 ELF2 LRBA SH3D19 FAM160A1 WWC2 CASP3 CCDC127 PDCD6 TPPP NSUN2 TRIO MYO10 ZFR RAI14 NIPBL DAB2 NNT PARP8 SLC38A9 IL6ST ZSWIM6 SREK1IP1 RAD17 MRPS27 HMGCR AP3B1 LNPEP PJA2 MAN2A1 TMED7 LMNB1 FBN2 ADAMTS19 RAPGEF6 SLC22A5 IRF1 SEC24A C5orf24 SPOCK1 CDC23 SLC35A4 PRELID2 PPARGC1B PDGFRB TCOF1 NDST1 SPARC LARP1 GEMIN5 ADAM19 TTC1 RANBP17 SH3PXD2B STC2 RNF44 GPRIN1 F12 WRNIP1 RIPK1 RREB1 HIVEP1 JARID2 ATXN1 ALDH5A1 BAK1 ITPR3 CDKN1A TBC1D22B PRICKLE4 MED20 CCND3 TAF8 PPP2R5D CUL7 PTK7 SRF CUL9 ZNF318 TMEM63B NFKBIE CDC5L ELOVL5 DST COL12A1 BCKDHB NT5E ZNF292 UBE2J1 MDN1 CDK19 REV3L DSE GOPC NHSL1 CCDC28A SYNE1 TMEM181 DYNLT1 IGF2R FAM120B PSMG3 LFNG CYTH3 DAGLB C7orf26 VWDE SNX13 IGF2BP3 OSBPL3 NOD1 AVL9 ELMO1 CDK13 HECW1 STK17A DBNL NUDCD3 CCM2 TNS3 GRB10 COBL VOPP1 CHCHD2 ZNF92 CCT6P3 KCTD7 CLIP2 GTF2I BAZ1B GTF2IRD2P1 STAG3L3 CLDN4 LIMK1 RHBDD2 YWHAG UPK3B CDK14 CYP51A1 ANKIB1 COL1A2 SMURF1 TRIM4 MCM7 AP4M1 GNB2 GIGYF1 CUX1 RELN ORC5 HBP1 LAMB1 CAPZA2 CTTNBP2 POT1 SND1 CALU FLNC TMEM209 CHCHD3 AKR1B1 CREB3L2 UBN2 CASP2 ARHGEF5 RARRES2 GIMAP6 SLC4A2 RBM33 ESYT2 ARHGEF10 MFHAS1 MTMR9 VPS37A PSD3 FAM160B2 BIN3 TNFRSF10B LOXL2 PTK2B SCARA3 ZNF395 EXTL3 RAB11FIP1 EIF4EBP1 BAG4 TACC1 ADAM9 RNF170 TGS1 CHD7 YTHDF3 CSPP1 NCOA2 ZFHX4 MMP16 NBN TP53INP1 PABPC1 RIMS2 TRPS1 RAD21 SQLE SLC45A4 ARC JRK ZC3H3 TSTA3 ZNF707 FAM83H TONSL C8orf33 KANK1 SMARCA2 GLDC NFIB MTAP B4GALT1 UBAP2 PIGO RUSC2 RNF38 DCAF10 GCNT1 PRUNE2 S1PR3 SEMA4D WNK2 FAM120A TRIM14 SEC61B MRPL50 FKTN SNX30 SLC31A1 ORM1 TNC PSMD5 GSN STOM RABGAP1 STRBP MAPKAP1 ZBTB34 RALGPS1 PTGES2 CIZ1 GOLGA2 COQ4 GLE1 SET ZER1 TBC1D13 LRRC8A GPR107 ASS1 ABL1 NUP214 FAM78A UCK1 TSC1 SURF1 WDR5 COL5A1 NACC2 GPSM1 SNAPC4 NOTCH1 UAP1L1 MAN1B1 ANAPC2 LARP4B BEND7 ZEB1 ARHGAP12 RET HNRNPF PRKG1 JMJD1C TET1 SUPV3L1 ANAPC16 USP54 SEC24C CAMK2G VCL KAT6B DLG5 PPIF LIPA KIF20B CPEB3 MYOF NOC3L CYP2C18 ENTPD1 LCOR RRP12 UBTD1 HOGA1 MARVELD1 GOT1 SCD HIF1AN PPRC1 NOLC1 PCGF6 SH3PXD2A ITPRIP DUSP5 TACC2 DMBT1 CHST15 ZRANB1 CTBP2 MKI67 PSMD13 RNH1 PHRF1 APBB1 RRP8 TMEM41B SNORA23 USP47 PLEKHA7 HPS5 HTATIP2 QSER1 HIPK3 AMBRA1 ARHGAP1 PACSIN3 DDB2 MADD SLC39A13 CELF1 NDUFS3 PTPMT1 TNKS1BP1 SSRP1 SLC43A3 SLC43A1 VPS37C DDB1 FADS2 FADS3 TUT1 EML3 B3GAT3 ZBTB3 MARK2 PLCB3 MAP4K2 EHD1 SAC3D1 CAPN1 DPF2 MAP3K11 OVOL1 EIF1AD RAB1B BRMS1 DPP3 SPTBN2 PTPRCAP PPP6R3 MYEOV DHCR7 NUMA1 RNF169 TSKU CLNS1A INTS4 ANKRD42 PRSS23 FAT3 CWF19L2 NPAT PCSK7 CEP164 IFT46 HINFP MCAM THY1 ARHGEF12 HSPA8 GRAMD1B VWA5A FLI1 APLP2 KDM5A RAD52 ITFG2 TSPAN9 TNFRSF1A NCAPD2 CHD4 ZNF384 PTPN6 LPCAT3 C1RL SLC2A3 ETV6 LRP6 AEBP2 RASSF8 TM7SF3 TMTC1 DENND5B FGD4 ALG10B ARID2 KANSL2 CCNT1 ADCY6 ARF3 TROAP MCRS1 TMBIM6 SMARCD1 ACVRL1 ACVR1B NR4A1 KRT81 ESPL1 SP1 COPZ1 ITGA5 ERBB3 PA2G4 ESYT1 PAN2 STAT2 BAZ2A R3HDM2 ARHGEF25 LRIG3 TMBIM4 FRS2 CCT2 RAB3IP PTPRB HAL APAF1 UTP20 TXNRD1 CORO1C ACACB ATP2A2 PPTC7 PLBD2 FBXW8 RFC5 PEBP1 TRIAP1 MLEC SPPL3 TMEM120B MLXIP SBNO1 GTF2H3 CCDC92 NCOR2 AACS EP400 IFT88 XPO4 SACS SPATA13 PARP4 SLC7A1 KATNAL1 N4BP2L2 PROSER1 SLC25A15 DGKH AKAP11 SETDB2 VPS36 TBC1D4 UCHL3 LMO7 MYCBP2 MBNL2 TM9SF2 COL4A1 COL4A2 CARS2 OSGEP CHD8 THTPA PSME2 RNF31 TINF2 COCH SRP54 SEC23A TRAPPC6B KLHL28 MGAT2 NEMF ARF6 FRMD6 FERMT2 SAMD4A SOCS4 PPM1A PRKCH SYNE2 MTHFD1 FUT8 ZFYVE26 SLC39A9 MAP3K9 SIPA1L1 ZFYVE1 LTBP2 JDP2 POMT2 STON2 SEL1L EML5 TTC7B TC2N TRIP11 SERPINA3 DICER1 CLMN PAPOLA CDC42BPB XRCC3 KIF26A AKT1 AHNAK2 PACS2 ADAM6 NIPA1 SNORD116-10 UBE3A TJP1 KLF13 OTUD7A THBS1 RPUSD2 RHOV TYRO3 MAPKBP1 TP53BP1 MAP1A PDIA3 SPG11 TMOD3 ADAM10 VPS13C RAB8B CA12 CSNK1G1 OAZ2 PIF1 DIS3L SMAD6 CLN6 ANP32A UACA BBS4 ADPGK NPTN LOXL1 ISLR ARID3B EDC3 ULK3 SCAMP5 PPCDC SNX33 UBE2Q2 PEAK1 BTBD1 ZNF592 AEN POLG ANPEP SEMA4B FURIN HDDC3 SLCO3A1 CHD2 LRRK1 CLCN7 TBL3 ZNF598 TRAF7 CCNF PDPK1 SRRM2 MGRN1 GLYR1 ABCC1 NOMO1 SMG1 SLC7A5P2 TNRC6A XPO6 CLN3 ATXN2L BOLA2 MVP CDIPT SEZ6L2 ASPHD1 DCTPP1 PRR14 FUS N4BP1 CHD9 HERPUD1 MMP15 CNOT1 CDH11 CDH5 CMTM3 CES2 CBFB SLC7A6 PDPR DDX19B ST3GAL2 VAC14 IST1 PSMD7 GLG1 MBTPS1 ANKRD11 TUBB3 SERPINF2 PAFAH1B1 ZZEF1 UBE2G1 KIF1C DHX33 DVL2 PHF23 NEURL4 ZBTB4 POLR2A KDM6B CYB5D1 PFAS MYH10 COX10 ZNF624 MPRIP ALKBH5 FLII TOP3A CCDC144B ULK2 DHRS7B TMEM11 RAB34 FLOT2 PHF12 GOSR1 ATAD5 LRRC37BP1 SUZ12 RFFL SLFN5 DHRS11 SYNRG PIP4K2B LASP1 MED1 CDK12 ORMDL3 MSL1 CASC3 TMEM99 KRT19 JUP NKIRAS2 STAT3 BRCA1 MPP2 TMEM101 ATXN7L3 SLC25A39 GRN GJC1 WNT3 HOXB3 ZNF652 COL1A1 SPAG9 NME1-NME2 NME2 VEZF1 MTMR4 USP32 PPM1D MED13 TLK2 TANC2 DCAF7 SMARCD2 ICAM2 DDX5 PRKCA BPTF PRKAR1A SLC9A3R1 NUP85 TSEN54 EXOC7 MGAT5B TNRC6C USP36 TIMP2 RNF213 NPTX1 LINC00482 SIRT7 DUS1L FASN YES1 LPIN2 RAB31 VAPA FAM210A GREB1L ABHD3 MIB1 DSG2 MAPRE2 EPG5 PSTPIP2 IER3IP1 CTIF TCF4 ZNF532 SERPINB2 ZNF516 BSG R3HDM4 SBNO2 MIDN DAZAP1 ATP8B3 MOB3A LMNB2 NCLN ZBTB7A SEMA6B KDM4B MLLT1 ARHGEF18 MAP2K7 RPS28 MUC16 DNMT1 KEAP1 SLC44A2 DNM2 CARM1 KANK2 RAB3D DHPS TNPO2 FARSA TECR SYDE1 NOTCH3 SLC27A1 JAK3 CCDC124 LSM4 SSBP4 ELL UPF1 SUGP2 ATP13A1 ZNF506 ZNF738 NUDT19 KCTD15 WTIP ZNF792 WDR62 ZNF260 ZNF420 ZNF585B ZNF585A SARS2 PSMC4 MAP3K10 AKT2 SERTAD1 SHKBP1 SNRPA EGLN2 AXL RPS19 MEGF8 BLOC1S3 ERCC1 RTN2 SIX5 SLC1A5 ZC3H4 DHX34 CYTH2 RPL18 ALDH16A1 RCN3 PRR12 BCL2L12 PNKP AKT1S1 ZNF578 NDUFA3 LENG8 NLRP2 PPP6R1 RPL28 ZNF628 ZNF581 ZNF580 ZNF460 ZNF304 ZNF587 ZNF417 ZNF8 TRIM28 NRSN2 CSNK2A1 ZNF343 VPS16 MAVS PANK2 PRNP CDS2 PLCB1 KIF16B RRBP1 ZNF133 SEC23B RALGAPA2 NAPB ZNF337 TM9SF4 ASXL1 PIGU GGT7 GSS EDEM2 EPB41L1 MYL9 MANBAL SRC PLCG1 HNF4A PLTP ARFGEF2 STAU1 RAB22A VAPB STX16 TAF4 LAMA5 CABLES2 OGFR TPD52L2 DNAJC5 NRIP1 TIAM1 URB1 MRPS6 SETD4 BRWD1 NDUFV3 CBS RRP1B PFKL FAM207A ADARB1 COL18A1 SLC19A1 LSS PCNT MICAL3 CDC45 MED15 SNAP29 HIC2 UBE2L3 PPIL2 PPM1F GGTLC2 MIR650 IGLL5 BCR GUSBP11 MMP11 SNRPD3 THOC5 PES1 PISD APOL6 RBFOX2 MYH9 TXN2 TRIOBP ANKRD54 MICALL1 TMEM184B GTPBP1 CBX6 SYNGR1 ATF4 XPNPEP3 EP300 XRCC6 CCDC134 SREBF2 TCF20 POLDIP3 ARFGAP3 FAM118A TUBGCP6 PLXNB2 PPP6R2 LMF2 ARSD NLGN4X CA5B RPS6KA3 EIF2S3 PDK3 POLA1 BCOR MED14 RBM10 CDK16 TBC1D25 SUV39H1 TIMM17B PRAF2 HUWE1 LAS1L ZMYM3 NONO TAF9B RBM41 COL4A5 AMMECR1 ALG13 SLC25A43 ELF4 MBNL3 HMGB3 SLC6A8 IRAK1 BRCC3 MTOR E2F2 ARID1A TMEM87B RGPD6 RGPD8 ANAPC1 TTN CAND2 CTDSPL LPP FGFR3 EVC VCAN UHRF1BP1 LIN28B SUN1 POM121C HIP1 PAXIP1 ASAP1 DENND4C ANKRD18A SLC44A1 DENND1A RAPGEF1 ZDHHC5 FAM111A IGHMBP2 SORL1 WNK1 MLF2 SCAF11 SLC38A1 DIP2B GOLGA3 STAT5B ITGB4 RPTOR PIK3R2 C19orf54 MTMR3 TNRC6B GNL3L | 1353 | miR-125b |

**Supplementary Table 4:** lncRNAs regulated by the two miRNAs.

| **Target lncRNAs** | **Total** | **miRNA** |
| --- | --- | --- |
| LINC00943 | 1 | miR-100-5p, miR-125b |
| LINC01641 SGO1-AS1 LINC00847 ST7-OT4 WAC-AS1 DLEU1 SDCBP2-AS1 LINC00943 | 8 | miR-100-5p |
| LINC01128 LINC01654 MIR29B2CHG LGALS8-AS1 CYP1B1-AS1 PCBP1-AS1 CYTOR TBC1D8-AS1 MIR4435-2HG PAX8-AS1 KLF7-IT1 ACVR2B-AS1 DUBR GATA2-AS1 TNK2-AS1 NOP14-AS1 TAPT1-AS1 DANCR STAG3L5P-PVRIG2P-PILRB LNCPRESS1 RNF139-AS1 GLIDR FAM27C ZSWIM8-AS1 RPARP-AS1 LINC01164 KCNQ1OT1 BDNF-AS MIR194-2HG NEAT1 LINC00943 N4BP2L2-IT2 ZFHX2-AS1 ST20-AS1 LINC00273 MAPT-IT1 LINC00667 PCAT18 PARD6G-AS1 LINC00661 LINC00261 PLAC4 PICSAR GUSBP11 INE1 LINC01278 XIST LINC00943 | 47 | miR-125b |
